# Bardet–Biedl Syndrome Presenting With Acute Kidney Injury Revealing Previously Undiagnosed Advanced Chronic Kidney Disease in a Young Adult: A Case Report

**DOI:** 10.1002/ccr3.73672

**Published:** 2026-10-02

**Authors:** Aqeel Abbas, Nouman Amjad, Amna Faheem Mufti, Muhammad Waqas, Minahil Iqbal, Hamza Ali, Amir Habib, Yahya Samadi, Muhammad Usman Haider

**Affiliations:** ^1^ Allama Iqbal Medical College Lahore Pakistan; ^2^ Gomal Medical College Dera Ismail Khan Pakistan; ^3^ Kateb University Kabul Afghanistan; ^4^ Geisinger Health System Wilkes‐Barre Pennsylvania USA

**Keywords:** acute kidney injury, Bardet–Biedl syndrome, case report, chronic kidney disease

## Abstract

Bardet–Biedl syndrome (BBS) is an autosomal recessive disorder characterized by obesity, rod‐cone dystrophy, polydactyly, developmental delay, renal dysfunction, and genitourinary abnormalities. We report the case of a 27‐year‐old female with previously undiagnosed BBS who presented with acute kidney injury precipitated by acute gastroenteritis and was subsequently found to have advanced chronic kidney disease. This case highlights how chronic kidney disease in BBS may remain unrecognized until adulthood in resource‐limited settings, emphasizing the importance of early diagnosis, regular renal surveillance, and multidisciplinary care to prevent irreversible renal damage.

## Key Clinical Message

Bardet–Biedl syndrome should be suspected in patients with retinal dystrophy, obesity, polydactyly, and renal dysfunction, particularly in consanguineous families. Early recognition and multidisciplinary surveillance may delay complications and improve quality of life.

**Abbreviations**
AKI: acute kidney injury
ALT: alanine transaminase
AST: aspartate transaminase
BMI: body mass index
BUN: blood urea nitrogen
CT‐KUB: computed tomography of kidneys, ureters, and bladder
Hb: hemoglobin
HCT: hematocrit
IQ: intelligence quotient
MCH: mean corpuscular hemoglobin
MCHC: mean corpuscular hemoglobin concentration
MCV: mean corpuscular volume
RBC: red blood cells
TLC: total leukocyte count
Wnt: wingless/integrated signaling pathway

## 1 Introduction

Bardet–Biedl syndrome (BBS) is a rare autosomal recessive hereditary disorder characterized by involvement of multiple systems. The typical features of the syndrome include *rod–cone* dystrophy that often leads to night blindness, central obesity, polydactyly, intellectual impairment, hypogonadism or genitourinary anomalies, and renal involvement. Because of its variable penetrance, the clinical presentation of BBS may differ significantly even within members of the same family [1, 2].

Although BBS occurs worldwide, its prevalence varies considerably between populations. In outbred populations of European descent, the disorder is estimated to affect between 1 in 125,000 and 1 in 160,000 individuals, whereas several genetically isolated communities show a substantially higher burden owing to founder mutations [1, 3]. The most striking example is the Faroe Islands, where a single founder splice‐site mutation in BBS1 (c.1091 + 3G > C) accounts for a prevalence as high as 1 in 3700 individuals; thirteen affected patients were identified among the islands' roughly 48,000 inhabitants, all tracing back to this shared variant [4].

By contrast, Pakistan has reported comparatively few cases of BBS, despite a burden of consanguinity that would, in principle, favor the expression of autosomal recessive disorders such as this one. Approximately half to two‐thirds of marriages in Pakistan are consanguineous, most commonly between first cousins, a pattern that increases the homozygosity of recessive disease alleles and underlies the founder mutations already identified in consanguineous Pakistani families with BBS [5, 6]. The relative paucity of reported Pakistani cases is therefore more likely to reflect underdiagnosis, stemming from limited access to ophthalmologic, nephrologic, and genetic testing services, than a genuinely low disease prevalence. This impression is reinforced by a recently published case series describing four affected individuals from two Pakistani sibling pairs, which illustrates both the marked intrafamilial variability of BBS and the diagnostic delay that can occur even when multiple family members are affected [7].

In this report, we present the case of a 27‐year‐old female from Pakistan who was born to first‐cousin parents. She exhibited typical features of BBS. She is one of three affected siblings in a family of six, reflecting the autosomal recessive inheritance pattern and the strong influence of consanguinity. This case adds to the limited literature available from Pakistan.

## 2 Case History/Examination

A 27‐year‐old female presented to the emergency department of Jinnah Hospital Lahore with a 7‐day history of vomiting and loose stools, gradual in onset and progressively worsening, associated with decreased urine output, fever, and abdominal pain.

Her past medical history was notable for a constellation of syndromic features recognized since childhood. She had intellectual disability from childhood and was considered a special child, though a specific syndromic diagnosis had never been formally established. She had developed night blindness at the age of 8, which progressed to complete blindness by age 19. She was overweight with a body mass index (BMI) of 29 and had primary amenorrhea. She had previously undergone cystostomy for a urethral stricture. Notably, two of her siblings were affected by a similar constellation of signs and symptoms. She had never previously undergone renal ultrasonography or biochemical evaluation, including assessment of estimated glomerular filtration rate (eGFR), as she had not sought medical attention for her syndromic features before this admission. Consequently, she had no prior diagnosis of chronic kidney disease and had never received renal replacement therapy at the time of presentation.

On general examination, the patient had syndromic features including bilateral polydactyly of the hands and feet (Figures 1 and 2), widely spaced eyes, and strabismus.

**FIGURE 1:**
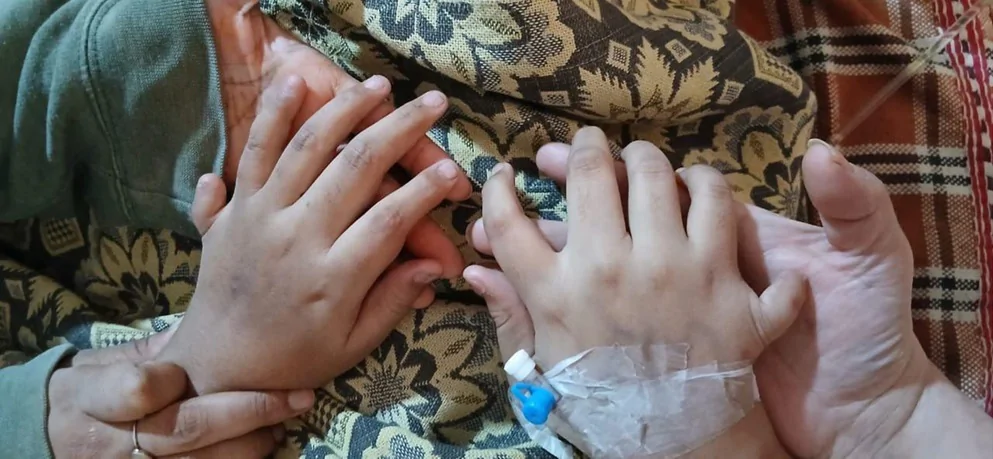
Bilateral polydactyly of the hands.

**FIGURE 2:**
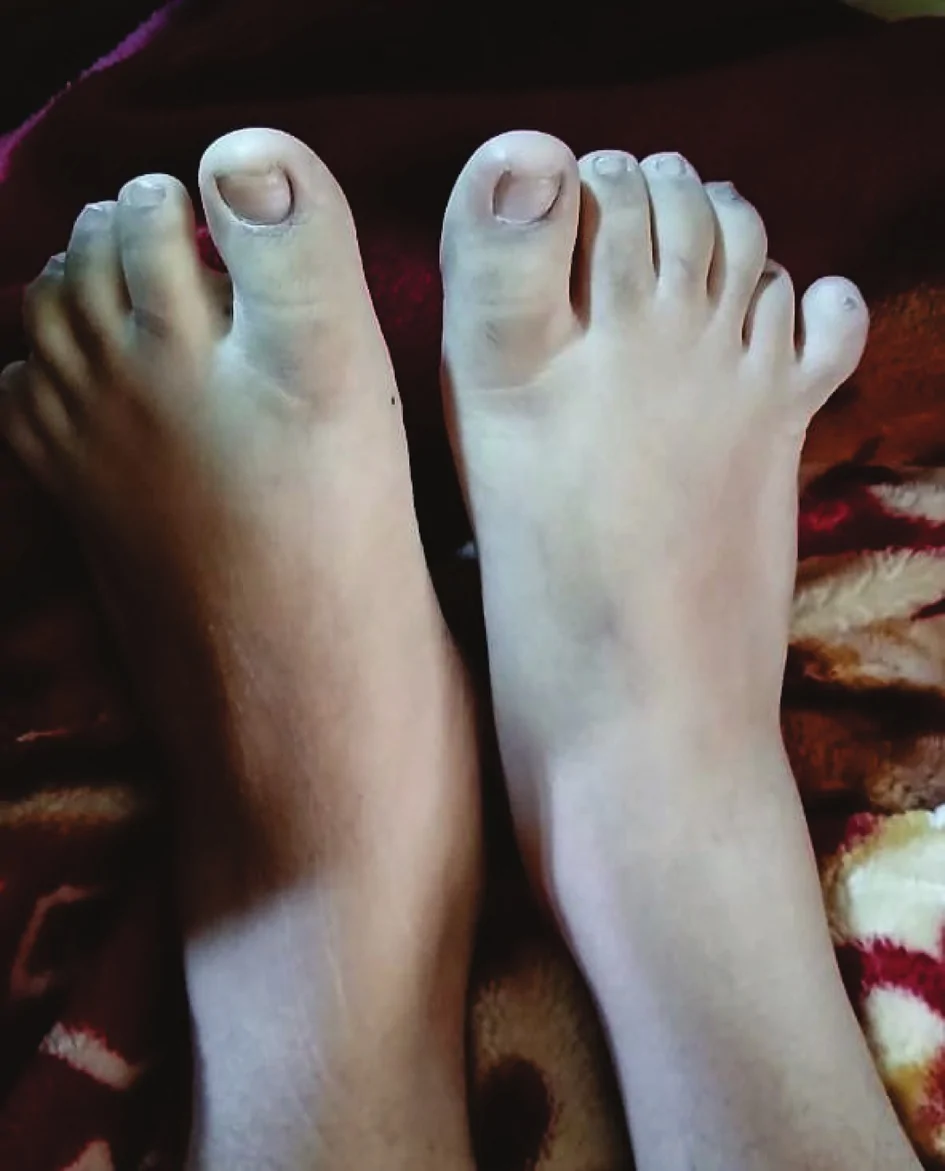
Bilateral polydactyly of the feet.

A blood sample was sent for baseline investigations. Key findings included macrocytic anemia, markedly elevated serum urea and creatinine consistent with severe renal impairment, elevated alkaline phosphatase, and a raised HbA1c with random blood glucose indicating poorly controlled diabetes mellitus. Liver function parameters and total protein were within normal limits. A summary of the principal laboratory abnormalities, together with their reference ranges, is provided in Table 1.

**TABLE 1:** Summary of major laboratory abnormalities.

Parameter	Patient value	Reference range	Interpretation
Hemoglobin	6.8 g/dL	12.0–15.5 g/dL	Severe anemia
Mean corpuscular volume (MCV)	112 fL	80–100 fL	Macrocytic
Serum urea	324 mg/dL	15–40 mg/dL	Markedly elevated
Serum creatinine	8.8 mg/dL	0.6–1.2 mg/dL	Markedly elevated
Alkaline phosphatase	480 U/L	44–147 U/L	Elevated
HbA1c	8.2%	< 5.7%	Poorly controlled diabetes mellitus
Random blood glucose	250 mg/dL	< 200 mg/dL (random)	Hyperglycemia

Abdominal ultrasonography revealed mild coarse liver parenchymal echotexture and mild splenomegaly. Bilateral renal cortices were mildly hypoechoic with increased echogenicity and reduced corticomedullary differentiation, suggesting parenchymal changes. CT‐KUB demonstrated bilaterally small kidneys (right: 83 × 27 mm; left: 83 × 33 mm) with lobulated, irregular cortices consistent with chronic renal parenchymal disease (Figure 3). Multiloculated fluid collections were identified in the subhepatic region, right iliac fossa, interloop regions, and bilateral adnexal regions. These imaging findings, taken together with the complete absence of any prior renal work‐up, confirmed that the patient had longstanding but previously undiagnosed chronic kidney disease, unmasked by the acute gastroenteritis episode.

**FIGURE 3:**
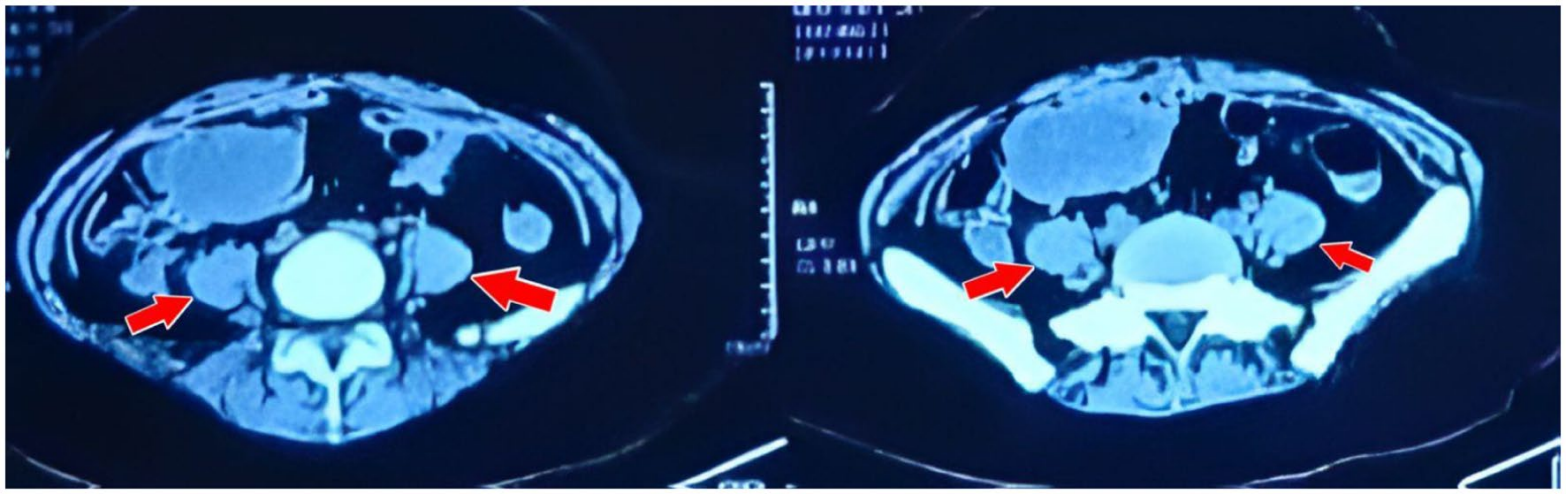
CT Scan demonstrated bilaterally small kidneys (right: 83 × 27 mm; left: 83 × 33 mm) with lobulated, irregular cortices.

## 3 Differential Diagnosis, Investigations and Treatment

The combination of retinal dystrophy progressing to blindness, chronic kidney disease, polydactyly, obesity, intellectual disability, primary amenorrhea, and a positive family history with similarly affected siblings raised the clinical suspicion of a hereditary ciliopathy. The principal differential diagnoses considered were Bardet–Biedl syndrome (BBS) and Alström syndrome. Bardet–Biedl syndrome characteristically presents with rod–cone dystrophy, obesity, polydactyly, renal anomalies, cognitive impairment, and hypogonadism—features closely mirroring this patient's presentation. Alström syndrome, while sharing retinal dystrophy and renal involvement, is not typically associated with polydactyly or intellectual disability, making it a less likely diagnosis. Given the classical constellation of findings, a clinical diagnosis of Bardet–Biedl syndrome was established. Genetic confirmation was not pursued due to resource limitations.

A diagnosis of acute kidney injury (AKI) on chronic kidney disease (CKD), precipitated by acute gastroenteritis, was made. The patient was treated with three sessions of hemodialysis, broad‐spectrum antibiotics, intravenous fluids, and antipyretics. Her condition improved over the subsequent days, with a notable increase in urine output. She was discharged with strict follow‐up advice.

## 4 Discussion

BBS is a disorder of primary ciliary function with substantial genetic heterogeneity; at least 26 genes have now been identified, most encoding proteins integral to ciliary assembly and intracellular trafficking [2, 8, 9]. Diagnostic criteria originally proposed by Beales et al. classified clinical features into primary criteria, comprising rod–cone dystrophy, polydactyly, obesity, learning disability, hypogonadism, and renal anomalies, and secondary criteria, comprising speech delay, developmental delay, diabetes mellitus, and dental anomalies among others, with a diagnosis requiring either four primary features or three primary features plus two secondary features [10]. A recent Inter‐European Reference Networks consensus statement has since revised these criteria to incorporate molecular diagnosis alongside the traditional clinical framework, reflecting the improved accessibility of next‐generation sequencing over the past decade [9]. Our patient fulfilled the primary diagnostic criteria, including rod–cone dystrophy, postaxial polydactyly, obesity, intellectual disability, genitourinary anomalies, and renal dysfunction [10]. The mean age of clinical diagnosis is reported at around 9 years, in contrast to our patient, who reached adulthood without ever receiving a formal diagnosis [3].

Visual impairment is typically the earliest and most consistent feature of BBS. Rod–cone dystrophy is a fully penetrant trait, estimated to affect over 90% of patients, and usually begins with night blindness in childhood before progressing to severe visual impairment or blindness by the mid‐teens to early adulthood [9]. Our patient's course, with night blindness at age eight progressing to complete blindness by age 19, closely mirrors this natural history. Obesity is likewise a near‐universal feature in adults with BBS and was also present in our patient [3, 9]. Primary amenorrhea, as seen in this case, is consistent with the hypogonadism reported in a substantial proportion of BBS patients, although fertility in affected women appears to be more often preserved than in affected men [9, 10]. Postaxial polydactyly, though not present in every patient, remains one of the most readily recognizable diagnostic features [3]. The presence of two similarly affected siblings in our patient's family underscores the autosomal recessive inheritance pattern of BBS and illustrates how consanguinity increases the likelihood of homozygosity for pathogenic variants within affected kindreds.

Renal involvement affects over half of patients with BBS and is one of the most clinically important determinants of long‐term prognosis, as kidney failure remains a leading cause of premature mortality in this population [2, 11]. The uro‐nephrological phenotype is highly variable, ranging from isolated structural anomalies, such as fetal lobulation, increased parenchymal echogenicity, and reduced corticomedullary differentiation, to overt cystic dysplasia and progressive decline in glomerular filtration [9]. A reduced urinary concentrating capacity, manifesting clinically as polyuria and polydipsia, is a common early indicator of underlying chronic kidney disease in BBS and reflects tubular dysfunction linked to disrupted ciliary signaling [9]. In the largest reported cohort to date, kidney failure requiring renal replacement therapy occurred in approximately 6% of adult and 5% of pediatric patients with BBS, and affected children have been observed to follow one of two broad trajectories, either developing severe chronic kidney disease early in life or remaining comparatively free of significant renal impairment through childhood [9, 11].

Our patient's course is notable in that she reached her third decade of life without a formal diagnosis of chronic kidney disease, despite imaging evidence of longstanding bilateral small kidneys with parenchymal scarring, indicating that renal injury had been accruing silently for years. This delayed recognition represents a missed opportunity for earlier surveillance that might otherwise have slowed the trajectory of her renal decline. The acute presentation itself, an episode of AKI superimposed on unrecognized CKD and precipitated by volume depletion from gastroenteritis, is a pattern that has only rarely been documented in adults with BBS, particularly from low‐ and middle‐income countries where systematic renal surveillance is not routinely available [7].

On a molecular level, disruption of ciliary signaling pathways, including the non‐canonical Wnt pathway, has been implicated in the cystogenesis and progressive nephron loss observed in BBS‐related kidney disease [8, 12, 13]. Genotype–phenotype studies from larger cohorts have further suggested that patients with variants in chaperonin‐like genes (BBS6, BBS10, BBS12) tend to develop more severe chronic kidney disease than those with BBS1 variants, an observation that may eventually help stratify renal surveillance intensity once genetic testing becomes more widely accessible in resource‐limited settings such as Pakistan [9].

Cardiovascular involvement, while less frequently reported, has been documented in a subset of patients with BBS and may include structural or functional cardiac abnormalities; our patient, however, showed normal cardiac function on echocardiography [14].

Given the multisystem nature of BBS, optimal long‐term management requires a multidisciplinary team spanning nephrology, ophthalmology, endocrinology, genetics, and cardiology, with clinical surveillance including regular blood pressure monitoring, annual ophthalmologic evaluation, and periodic assessment of renal function and metabolic parameters [9, 10]. Management of kidney and urinary tract disease in BBS does not otherwise differ from standard nephrology practice and should follow established guidelines for the evaluation and management of chronic kidney disease [9]. Should progression to kidney failure occur, renal transplantation remains a viable option with outcomes comparable to the general population, although coexisting obesity may complicate both the pre‐transplant work‐up and post‐transplant metabolic management [9].

Genetic counseling remains central to comprehensive BBS care, and families should be informed about recurrence risk and the implications of the disorder's marked genetic heterogeneity [12]. Although no disease‐modifying therapy currently exists for the renal or retinal manifestations of BBS, early diagnosis, structured surveillance, and multidisciplinary follow‐up can meaningfully improve quality of life and help delay the onset of irreversible complications.

Reports of BBS from Pakistan remain scarce relative to the country's population size and consanguinity burden, which likely reflects underdiagnosis rather than a genuinely low disease prevalence. Table 2 summarizes the published Pakistani cases identified in the literature to date, alongside the present report.

**TABLE 2:** Published cases of Bardet–Biedl syndrome from Pakistan.

Author (year)	Location	Patients (*n*)	Consanguinity	Key clinical features
Khan et al. (2019) [15]	Pakistan	1	Not reported	Polydactyly, obesity, retinal dystrophy, developmental delay
Muzammal et al. (2019) [6]	Dera Ismail Khan	2 families	Consanguineous	BBS9 founder mutation (c.299delC); retinopathy, obesity, polydactyly, renal dysfunction
Babar et al. (2024) [16]	Peshawar	1	Not reported	Osmotic symptoms, new‐onset diabetes mellitus, polydactyly, retinal dystrophy
Ikram et al. (2025) [17]	Peshawar	1 (12‐year‐old boy)	Nonconsanguineous	Polydactyly, syndactyly, central obesity, retinitis pigmentosa, diabetes mellitus
Jawed et al. (2025) [18]	Karachi	1	Consanguineous	Polydactyly, developmental delay, endocrine abnormalities
Aziz et al. (2026) [7]	Multan	4 (2 sibling pairs)	1 pair consanguineous, 1 nonconsanguineous	Marked intrafamilial variability; retinal dystrophy, obesity, polydactyly, renal/urinary findings
Irshad et al. (2026) [19]	Islamabad	3	Not reported	Polydactyly, short stature, developmental delay, renal/hepatic/genital ultrasound abnormalities
Present case	Lahore	1 (27‐year‐old female)	Consanguineous	Rod–cone dystrophy, polydactyly, obesity, intellectual disability, primary amenorrhea; AKI on previously undiagnosed advanced CKD

Taken together, these reports share several recurring features: most patients originate from consanguineous unions, present in childhood or adolescence with retinal and developmental features, and are diagnosed clinically rather than genetically because of limited access to molecular testing. Renal involvement, when specifically assessed, has been variably reported, and the present case appears to be among the few from Pakistan in which unrecognized chronic kidney disease progressed to acute kidney injury in adulthood before the underlying syndromic diagnosis was ever established. Greater clinical awareness of BBS among pediatricians, ophthalmologists, and family physicians in Pakistan, together with more systematic renal surveillance of children presenting with the syndrome's cardinal features, could allow earlier diagnosis and potentially forestall the kind of delayed, acute presentation described here.

## 5 Conclusion

BBS is characterized by classical features such as obesity, hypogonadism, intellectual disability, polydactyly, renal impairment, and retinitis pigmentosa. Progressive renal dysfunction is the leading cause of death in affected individuals. Due to seemingly unrelated symptoms, the condition is often underdiagnosed. Increasing awareness of genetic disorders is important, as multidisciplinary care and regular follow‐up can significantly improve quality of life and decrease complications. Genetic counseling for families can highlight the risk of recurrence in future children and convey the importance of screening healthy relatives for potential complications.

## Author Contributions

**Aqeel Abbas:** conceptualization, formal analysis, supervision. **Nouman Amjad:** investigation, methodology, formal analysis. **Amna Faheem Mufti:** visualization, formal analysis, supervision. **Muhammad Waqas:** conceptualization, visualization, data curation, supervision. **Minahil Iqbal:** investigation, validation, visualization, writing – review and editing, project administration. **Hamza Ali:** writing – original draft, data curation, visualization. **Amir Habib:** investigation, validation, formal analysis. **Yahya Samadi:** funding acquisition, project administration, validation. **Muhammad Usman Haider:** resources, investigation, writing – review and editing.

## Funding

The authors have nothing to report.

## Ethics Statement

Ethical approval was waived by Jinnah Hospital Lahore as this is a case report and does not involve a research study.

## Consent

Written informed consent was obtained from the patient and her father for participation in this case report, in accordance with the journal guidelines.

## Conflicts of Interest

The authors declare no conflicts of interest.

## Data Availability

The data that support the findings of this study are available from the corresponding author upon reasonable request.
